# Evaluating the UE‐ATR Checklist: Nuanced Attribution in Unsuccessful Therapeutic Outcomes

**DOI:** 10.1002/cpp.70091

**Published:** 2025-05-28

**Authors:** Sanne T. L. Houben, Anna C. P. Backus, Suzanne Hermans, Harald Merckelbach, Brechje Dandachi‐FitzGerald

**Affiliations:** ^1^ Department of Clinical Psychological Science Maastricht University Maastricht the Netherlands; ^2^ Faculty of Psychology Open University Heerlen the Netherlands

**Keywords:** psychotherapy, therapeutic side effects, UE‐ATR checklist, unwanted effects

## Abstract

Unwanted events in psychotherapy can hinder treatment, yet clinicians overlook them and tend to attribute treatment stagnation mainly to patient‐related factors. The unwanted events–adverse treatment reaction (UE‐ATR) checklist was developed to encourage a more balanced reflection on treatment difficulties, but its effectiveness remains unclear. This study investigated whether the UE‐ATR checklist enables clinicians to allocate treatment difficulties in a more nuanced way across various contributing factors. Clinicians and psychology students (*N* = 104) were randomly assigned to either use the UE‐ATR (*n* = 59) or not (*n* = 45) while reviewing a case vignette of a patient who experienced unwanted events during therapy and treatment stagnation. They allocated responsibility for suboptimal treatment outcome across five factors: the patient, the therapist, the treatment method, the patient's pathology or other circumstances. Attribution was analysed using the Herfindahl–Hirschman index (HHI), where higher scores indicate a monocausal and lower scores reflect a multicausal view. No significant differences were found between the conditions. Although most users found the checklist clinically useful, this positive perception did not lead to a more balanced perspective on the causes of unwanted events. Although the UE‐ATR checklist can support clinical reflection, additional training is necessary to maximize its effectiveness.

**Summary**

- Unwanted events during therapy are often underestimated and primarily attributed to patient‐related factors, which may hinder effective treatment.
- The UE‐ATR is a potential tool to support more balanced reflection on unwanted events during therapy.
- Additional training is needed to help clinicians utilize tools like the UE‐ATR.

## 1 Introduction

Psychotherapy has been shown to alleviate psychological symptoms across a range of psychopathological conditions, including depression and anxiety disorders (Cuijpers et al. 2024; Wakefield et al. 2021). Still, unwanted events (UEs) can occur during or alongside psychotherapy (Klatte et al. 2023; Strauss et al. 2021). UEs are typically defined as lack of improvement or worsening of symptoms (Hardy et al. 2017; Klatte et al. 2023). However, UEs emerging during or after treatment can go beyond symptom deterioration or non‐response, including, for example, negative well‐being after a therapy session, strained relationships or stigmatization (Crawford et al. 2016; Cuijpers et al. 2018; Klatte et al. 2023; Linden 2013). Monitoring such UEs is important in clinical practice, as it allows both the patient and the clinician to evaluate the benefit–risk ratio, consider the treatment's burden and determine whether any modifications could support the therapeutic process and overall treatment goals.

In general, clinicians face challenges in recognizing UEs during therapy (Werbart et al. 2019). First, there is no clear and consistent definition of UEs, which contributes to a lack of consensus on what constitutes such events and, consequently, what clinicians should monitor (Batic and Hayes 2020; Klatte et al. 2023; Parry et al. 2016; Paveltchuk et al. 2022). Second, in psychotherapy trials, monitoring UEs is often not included or limited to serious adverse events, leaving the true prevalence of these events and their potential causal relation to the therapeutic intervention unclear (Jeckel et al. 2024; Klatte et al. 2023). Third, when clinicians do recognize UEs related to therapy, they may hesitate to address them due to concerns about being perceived as ineffective or lacking skill (Linden 2013). Fourth, clinicians perceive certain UEs as an unavoidable part of therapy that might even be necessary for positive outcomes and therefore ultimately view them as positive (Jonsson et al. 2016; Moritz et al. 2019).

However, patients often experience UEs as more intrusive than clinicians perceive them to be (Hatfield et al. 2010). Also, patients may also report fewer positive treatment effects after experiencing UEs (Moritz et al. 2019). This, however, is not universally the case, as another study suggests a more complex relationship (Verkooyen et al. 2024), indicating the need for further research in this area. Identifying UEs and determining whether they result from therapy require clinical judgement, which presupposes skills from clinicians (Moritz et al. 2015; Schermuly‐Haupt et al. 2018). However, and related to the reasons listed above, such skills are often underemphasized in training and education (Curran et al. 2019; Westin and Rozental 2024). Monitoring tools can be valuable in assisting clinicians with identifying UEs. The unwanted events–adverse treatment reaction (UE‐ATR; Linden 2013) checklist is one such tool, designed to help clinicians recognize, categorize and document these events. The UE‐ATR is based on clinical experience and pharmacotherapy research and includes several UEs such as lack of clear treatment results, non‐compliance of the patient and emergence of new symptoms. Using the UE‐ATR supports the identification of UEs by providing a structure so that clinicians do not overlook certain aspects. Although this tool is recommended for clinical training (Nolan et al. 2004; Schermuly‐Haupt et al. 2018), its added value has yet to be empirically explored.

The aim of this study was to assess whether the UE‐ATR checklist serves as a valuable tool for clinicians in recognizing, identifying and classifying UEs during treatment. Specifically, we examined whether its use would foster a more nuanced and balanced evaluation of suboptimal treatment outcomes. Participants were presented with a case vignette describing a patient who experienced UEs during therapy, ultimately leading to treatment stagnation. In line with the UE‐ATR's objectives, we hypothesized that participants who received the checklist alongside the vignette would attribute treatment stagnation to multiple causes more frequently than those who did not receive the checklist.

## 2 Method

### 2.1 Transparency and Openness

We report how we determined our sample size, all data exclusions, manipulations and measures in the study, and the study follows JARS (Appelbaum et al. 2018). Research materials, data and analysis code are available at the Open Science Framework (https://osf.io/3dsz8/). Data were analysed using SPSS. This study was preregistered prospectively before data were collected; see https://osf.io/6ygtb.

### 2.2 Participants

Preregistration included a 2 (UE‐ATR checklist: yes vs. no) × 2 (expertise: students vs. professionals) mixed design and power analysis. An a priori power analysis using G*Power (Faul et al. 2007) with a medium effect size of 0.25, a power of 0.80 and a standard alpha error probability of 0.05 indicated that a total sample of 180 participants was required to assess both the added value of the UE‐ATR and the effect of professional expertise. The study was conducted as a paper‐and‐pencil task in lab (see below) but was delayed because of COVID‐19. As work and study activities shifted online, we chose not to burden students and clinicians with an online study. Because of these COVID‐related difficulties in recruitment, the sample consisted of 110 participants. To avoid an underpowered study, as indicated by a post hoc power analysis, we narrowed our focus to assessing whether the UE‐ATR checklist aids in recognizing factors influencing therapy outcomes, rather than examining the effect of expertise (i.e., students vs. professionals). Consequently, we employed an independent sample *t*‐test, yielding a post hoc power of 0.71.

Participants were recruited during classes (students) or via the professional networks of S.H. and B.D. (professionals). Of these, six participants were excluded, because therapeutic success was evaluated with an implausible rating of 10 (*n* = 1; see below), control questions were incorrectly answered (*n* = 7), or points allocated to the cause of failure did not add up to 100 (*n* = 4; see below). The final sample consisted of 104 participants (*n*_students_ = 37, *n*_professionals_ = 67, 88 women (84.6%), *M*_age_ = 31.18, SD = 11.36). Seventy‐one participants (68.3%) had been in treatment themselves and/or knew someone close to them who was. See Table 1 for the functions of participants. On average, the professionals had 10.41 years of clinical experience (SD = 8.95; range, 0–40).

**TABLE 1:** Background of sample.

Function	*n* (%)
Psychologist	30 (44.8)
Health care psychologist	18 (26.9)
Health care psychologist in training	5 (7.5)
Psychotherapist	2 (3.0)
Psychotherapist in training	2 (3.0)
Clinical psychologist and psychotherapist	2 (3.0)
Clinical neuropsychologist in training	1 (1.5)
Students	37 (35.6)

### 2.3 Materials

#### 2.3.1 Case Vignette

Before data collection, a pilot study was conducted with five mental health professionals to evaluate the case vignette. They had to rate the case vignette on credibility (0–10) and then evaluate whether the instruction was clear (yes/no), how long it took to read the case vignette and answer the questions and whether they had any recommendations for improvement. Four participants took between 15 and 30 min and one participant took between 0 and 15 min to read the case vignette. The case vignette had a mean credibility of 8.75 (SD = 0.96), which was found acceptable.

The case vignette concerned Peter, a 45‐year‐old police officer who reports to an outpatient mental health institution with trauma‐related and depressive complaints. Peter experienced UEs during his treatment. For example, the clinician showed up late, which made Peter feel unimportant, and he experienced distress after an EMDR session. Trauma‐related symptoms decreased, but his depressive mood did not, and Peter started to feel hopeless. After CBT, his mood improved slightly. Eventually, Peter requested to end the treatment. The full description of the case vignette can be found at the OSF.

#### 2.3.2 UE‐ATR Checklist

The UE‐ATR checklist (Linden 2013) is a checklist to guide clinicians in recognizing 16 UEs and deciding whether these can be attributed to the therapy (i.e., adverse treatment reaction). The UEs are classified, among others, as lack of clear treatment results, prolongation of treatment and deterioration of symptoms. The clinician can indicate whether the UEs are present or not. If so, the clinician must assess three factors: the context in which the UEs developed, its relation to the treatment and its severity. To assist in this process, the clinician can refer to a rating glossary. For context, the clinician can choose from eight categories: diagnostic procedures, theoretical orientations, selection of the focus of treatment, treatment procedures, sensitization processes, disinhibition processes, treatment effects or therapist–patient relationship. The relationship to treatment is rated on a 5‐point scale (1 = *unrelated*; 2 = *probably unrelated*; 3 = *possibly related*; 4 = *probably related*; 5 = *related*), and severity is rated on a 5‐point scale (1 = *mild, without consequences*; 2 = *moderate, some impact*; 3 = *noticeable, minor consequences*; 4 = *severe, significant consequences*; 5 = *extremely severe, requiring hospitalization or life‐threatening*). Participants in the current study were instructed to use the UE‐ATR checklist in evaluating the case of Peter and to use it as a basis for deciding how successful his treatment had been.

#### 2.3.3 Evaluation Questionnaire

Participants first answered questions about their biographical information, current job (professionals) and their own experience with psychological treatment. Three control questions were included (‘What is Peter's profession?’ ‘What treatment(s) does Peter receive?’ and ‘Is Peter going back to work?’). Participants were asked to rate the success of the therapy in this case on a scale from 0 to 10 (anchors: 0 = *not successful*; 10 = *very successful*). Additionally, they allocated points (ranging from 0 to 100) to various potential causes of the therapy's (partial) failure, with higher scores indicating a stronger perceived causal impact. The listed factors included the therapist, the chosen treatment method, the patient, the patient's disorder and other circumstances. The total assigned points had to sum up to 100. An attention question (i.e., ‘If I read this question correctly, I score 0 here’) was included as well. To determine whether participants had a balanced view on the causes of partial failure (considered multiple causes), we calculated the Herfindahl–Hirschman index (HHI; Namkoong and Henderson 2014). This index ranges from 0 to 1 and is used in microenomics to examine whether there is a monopoly (i.e., one cause), oligopoly (i.e., a few equal causes) or a well‐spread range of providers (i.e., nuance). A high index (i.e., closer to 1) indicates a greater concentration on one cause (i.e., one cause receives most points), reflecting a monocausal perspective on therapeutic failure. A low index (i.e., closer to 0) indicates a more evenly distributed range of causes, reflecting a more nuanced, multicausal perspective on therapeutic failure. Participants who had received the UE‐ATR checklist were additionally asked to evaluate the effectiveness of the checklist in assessing therapy outcome and whether the checklist could be a useful tool in clinical practice.

#### 2.3.4 Procedure

Students were seated in a quiet room and given an envelope with the information letter, informed consent, instructions, the case vignette with (out) the UE‐ATR checklist and the evaluation questionnaire. Professionals were given a sealed envelope with the same materials and were asked to read it in a quiet place and at a quiet time. Participants evaluated the case vignette either with the assistance of the UE‐ATR checklist (*n* = 59) or without it (control condition; *n* = 45). The allocation was double‐blind.

The participants returned the material in a closed envelope. Participants were briefed after all data had been collected. Ethical approval was obtained from the standing Ethical Review Committee of the Faculty of Psychology and Neuroscience (ERCPN‐207_09_04_2019).

## 3 Results

### 3.1 Therapeutic Success

An independent samples *t*‐test revealed no differences in therapy success ratings between the group with the checklist (*M* = 7.05, SD = 1.09) and the group without the checklist (*M* = 7.01, SD = 1.13); *t*(102) = 0.18, *p* = 0.856, Cohen's *d* = 0.036.

### 3.2 UE‐ATR Checklist and HHI

Our dependent variable was the degree of nuance in allocating causes to the suboptimal treatment outcome, which was addressed by comparing the HHI indices of the checklist group (*M* = 0.362, SD = 0.14) and the no‐checklist group (*M* = 0.363, SD = 0.15). An independent samples *t*‐test revealed no significant difference between the groups, *t*(102) = 0.03, *p* = 0.975, Cohen's *d* = 0.006. To further assess the strength of evidence for the null hypothesis, a Bayesian independent samples *t*‐test was conducted using a default Cauchy prior (scale = 0.707). The analysis produced a Bayes factor (*BF*_10_ = 0.209), providing moderate evidence for the null hypothesis, suggesting comparable HHI ratings across both groups. When specifically calculating the HHI for clinicians (checklist group, *n* = 39, *M* = 0.357, SD = 0.13; no‐checklist group, *n* = 28, *M* = 0.359, SD = 0.16), no statistical significance difference emerged, *t*(65) = 0.05, *p* = 0.960, Cohen's *d* = 0.155.

To explore how participants allocated points across the five potential causes of therapeutic failure, we calculated the mean scores for each cause (see Table 2). A 2 (condition: checklist vs. no‐checklist) × 5 (causes) repeated measures ANOVA revealed no significant main effect of condition (*F*(1, 102) = 0.72, *p* = 0.397) and no significant interaction between condition and causes (*F*(4, 102) = 0.41, *p* = 0.800). However, there was a significant main effect of causes (*F*(4, 102) = 32.52, *p* < 0.001, *η* ^2^ = 0.241). Post hoc Bonferroni‐corrected comparisons indicated that *Therapist* and *Other circumstances* were given significantly more causal weight than *Patient*, *Disorder* and *Treatment method*. No significant differences were found between *Therapist* and *Other circumstances* or among the other three causes.

**TABLE 2:** Mean scores (SD) per cause in the checklist versus no‐checklist condition.

Causes	Checklist (*n* = 59)	No‐checklist (*n* = 45)	Post hoc Bonferroni comparisons
1. Therapist	33.86 (20.34)	34.22 (20.03)	1 > 2, 3, 4
2. Treatment method	11.10 (16.08)	11.67 (13.78)	2 < 1, 5
3. Patient	16.49 (13.32)	13.44 (9.88)	3 < 1, 5
4. Disorder	13.56 (14.14)	12.11 (13.92)	4 < 1, 5
5. Other circumstances	25.64 (15.45)	28.56 (18.08)	5 > 2, 3, 4

### 3.3 Evaluation UE‐ATR

Participants who received the UE‐ATR checklist were asked with open‐ended questions to evaluate the effectiveness of the checklist in assessing therapy outcome and whether the checklist could be a useful tool in clinical practice. Based on their comments, we coded their evaluation (i.e., positive, positive but complicated, negative, did not use the checklist and more information needed).

A slight majority of 34 participants (57.6%) were positive about its effectiveness and stated, for example, that it provides an overview of factors to be considered. Eight participants (13.6%) were positive but found the tool complicated. To illustrate, they stated that the checklist was too extensive, or it was unclear how to use the checklist. Five participants (8.5%) were negative and found the checklist too cumbersome or difficult to use. Six participants (10.2%) did not use the checklist. Another six participants (10.2%) needed more information to decide whether the checklist was effective. They could see potential in using the checklist but had remaining questions about, for example, therapeutic experience and number of therapeutic sessions.

Regarding its clinical merit, 37 participants (62.7%) stated that the checklist would be useful in clinical practice. Participants noted that the checklist provides focus and encourages broader consideration of factors, reducing the risk of clinicians prioritizing personally salient symptoms. Sixteen participants (27.1%) were not convinced of its clinical value and stated that the checklist should have clearer instructions or could be shortened. Six participants (10.2%) did not see its clinical value, because it is labour intensive and not user friendly or simply because there are enough checklists in mental health care.

## 4 Discussion

The primary objective of this study was to empirically examine the usefulness of the UE‐ATR and whether its use would lead to a more nuanced assessment of a not (fully) successful therapy. Although we expected that participants who employed the UE‐ATR checklist would be more nuanced in attributing partial therapy failures to causes, no statistically significant difference with the no‐checklist control condition emerged. Participants in both conditions were rather balanced (i.e., HHI of 0.32) in their cause allocation. Thus, our results do not support the intended goal of the UE‐ATR checklist—enhancing clinicians' awareness of the numerous factors contributing to UEs and ultimately fostering a more informed evaluation of potential causes (Schermuly‐Haupt et al. 2018).

Interestingly, in both conditions, the therapist and other circumstances received the highest causal weight estimates. Participants assigned greater responsibility to the clinician than to patient‐related factors, which contrasts sharply with the common belief that clinicians tend to attribute therapy failure to patients (e.g., Murdock et al. 2010; Walfish et al. 2012). Notably, this assumption has not consistently held up to empirical scrutiny in other studies (e.g., Dandachi‐FitzGerald et al. 2022).

Remarkably enough, although we found no evidence that the checklist provides its users with a more balanced view on therapy failure, many deemed the checklist to be effective and valuable in clinical practice. However, a few participants also pointed to the lack of clear instructions and the complexity of the instrument, leading some to believe that the checklist had no added value. Arguably, additional information and training on the checklist's application are needed before its added value can be properly evaluated (Schermuly‐Haupt et al. 2018). Only through this approach, clinicians might become better equipped to identify unwanted therapeutic effects and understand their origins (see also Castonguay et al. 2010).

The current study has several limitations. First, we did not control to what extent participants in the checklist group in fact used this checklist to evaluate the case vignette. In retrospect, we might have given these participants more detailed instructions. On the other hand, the checklist is meant for clinical practice and when its use is not straightforward, one might question, at this stage, its practical value. Second, the case vignette may not have clearly conveyed an unsuccessful therapy outcome. Although it was designed to represent a suboptimal result, participants in both the UE‐ATR checklist and the control condition rated therapy's success relatively high (i.e., 7/10). In retrospect, it might have been better to use a case vignette that represents a more obvious therapeutic failure. On the other hand, the UE‐ATR is most needed in cases that are ambiguous and require clarification. Third, the case vignette depicted a fictional scenario. Although experienced clinicians in our pilot study rated it as highly realistic, it may still be less compelling than a situation in which participants evaluate real patients and the UEs that occurred during therapy they personally administered.

The current study did not find evidence that the UE‐ATR checklist promotes a more nuanced perspective on therapy that is not (fully) successful compared to not using the checklist. However, clinicians still perceive it as clinically valuable. Before its effectiveness can be empirically evaluated, it is crucial to provide clear and more user‐friendly instructions on how to apply the checklist correctly. Without ensuring that clinicians fully understand and engage with the tool as intended, any assessment of its true value remains premature. Until these criteria are met, the jury is still out, even if clinicians subjectively feel that the checklist benefits them. Future research should refine its evaluation by (1) incorporating a condition where clinicians assess their own therapy cases and (2) enhancing the checklist's instructions, alongside a manipulation check, to ensure consistent and proper use. These improvements could offer deeper insights into the clinical merit of the UE‐ATR checklist.

## Author Contributions

**Sanne T. L. Houben:** conceptualization, methodology, project administration, resources, supervision, writing – original draft. **Anna C. P. Backus:** writing – review and editing. **Suzanne Hermans:** conceptualization, resources, writing – review and editing. **Harald Merckelbach:** conceptualization, methodology, writing – review and editing. **Brechje Dandachi‐FitzGerald:** conceptualization, methodology, formal analysis, project administration, resources, supervision, writing – review and editing.

## Conflicts of Interest

The authors declare no conflicts of interest.

## Data Availability

Research materials, data and analysis code are available at the Open Science Framework (https://osf.io/3dsz8/).
